# Insect pollination: an ecological process involved in the assembly of the seed microbiota

**DOI:** 10.1038/s41598-020-60591-5

**Published:** 2020-02-27

**Authors:** Alberto Prado, Brice Marolleau, Bernard E. Vaissière, Matthieu Barret, Gloria Torres-Cortes

**Affiliations:** 1 Escuela Nacional de Estudios Superiores, Unidad Juriquilla, UNAM, Querétaro, México; 2 Institut de Recherche en Horticulture et Semences, Agrocampus-Ouest, INRAE, Université d’Angers, SFR4207 QuaSaV, 49071 Beaucouzé, France; 3 Institut national de recherche pour l’agriculture, l’alimentation et l’environnement, UR 406 Abeilles et Environnement, F 84914 Avignon cedex 9, France

**Keywords:** Community ecology, Microbiology, Microbial ecology, Microbiome

## Abstract

The assembly of the seed microbiota involves some early microbial seed colonizers that are transmitted from the maternal plant through the vascular system, while other microbes enter through the stigma. Thus, the seed microbiota consists of microbes not only recruited from the plant vascular tissues, but also from the flower. Flowers are known to be a hub for microbial transmission between plants and insects. This floral-insect exchange opens the possibility for insect-transmitted bacteria to colonize the ovule and, subsequently, the seed to pass then into the next plant generation. In this study, we evaluated the contribution of insect pollination to the seed microbiota through high-throughput sequencing. Oilseed rape (OSR) flowers were exposed to visits and pollination by honey bees (*Apis mellifera*), red mason bees (*Osmia bicornis*), hand pollinated or left for autonomous self-pollination (ASP). Sequence analyses revealed that honey bee visitation reduced bacterial richness and diversity in seeds, but increased the variability of seed microbial structure, and introduced bee-associated taxa. In contrast, mason bee pollination had minor effects on the seed microbiota. Our study provides the first evidence that insect pollination is an ecological process involved in the transmission of bacteria from flowers to seeds.

## Introduction

In nature, plants live in close association with a diversity of micro- and macro-organisms, both within and outside their tissues. Microbes may play beneficial roles in plant growth and development, positively affecting plant biomass or disease resistance^1–3^. Although numerous studies have focused on microbial assemblages associated with different plant organs^4,5^, little is known about tripartite interactions between plants, their microbiomes and other multicellular organisms, such as pollinators. Insect visitors acquire and deposit microorganisms onto flower surfaces during nectar and pollen collection^6–9^, thus shape the flower microbiota^10–12^. These flower-associated microbes are mainly fungi, followed by bacteria^10^. A recent study has shown that these flower inhabitants can act as intermediaries of plant-pollinator communication; bees innately avoid flowers inhabited by bacteria but are not deterred by yeasts^13^. Moreover, microorganisms transported by insects may influence plant-pollinator interactions; this is the case of yeasts transported by ants that change nectar composition^8^. Furthermore, since the flower microbiota serves as one of several inocula for the plant ovule and, hence, for the seed^14^, it is possible that by affecting the microbial community of the flower (including pollen), pollinators could modify the seed microbiota.

The role of insect vectors in the dispersal of bacteria and fungi to roots, stems, leaves, flowers, and fruits is well documented^15,16^, while their role in the microbial assembly of the seed has yet to be described. During seed-to-seed development, some early microbial seed colonizers are transmitted from the mother plant to the ovule through the vascular system (internal transmission) while others colonize the pistil and the ovary to finally reach the seed (floral transmission)^14,17^. The floral pathway for the seed tissue colonization is less specialized than the vascular (internal) pathway. Indeed, plant-pathogenic bacteria can colonize both host and non-host seeds via the floral pathway^18^. Floral-to-seed transmission has been achieved by depositing microbes onto flowers^17,18^. Other microbes are incorporated into the seed at later stages, via external transmission, due to the contact with microorganisms present on fruits, flowers or threshing residues^19^. Thus, the assembly of the seed microbiota is a complex process, including microbes recruited not only from the vascular tissue of the plant, but also from the floral microbiota. By affecting floral traits, microorganisms inhabiting the flower can have beneficial or detrimental consequences for the reproductive success of the plant^10,13,20,21^. However, it is unknown whether microbes inhabiting the flower can be incorporated via the floral pathway and affect seed traits. It is, therefore, important to understand the drivers in the assembly of seed microbiota.

Previous community-profiling approaches performed on the seed microbiota of various plant species have identified a range of bacterial taxa that could potentially be insect-transmitted^14,22–30^. For instance, bacterial taxa affiliated to the *Enterobacteriaceae*, such as the ubiquitous *Pantoea agglomerans*, are found both in flowers and seeds as well as insect visitors, such as the honey bee *Apis mellifera*^28,31^. In addition, another *Enterobacteriaceae* species, *Rosenbergiella nectarea*, isolated from the nectar of diverse plant species^32^, has also been detected in seeds^33^, suggesting a possible bacteria transmission to the seed through the floral pathway by insect pollinators. However, it is currently unknown if insect pollinators participate in shaping the structure of the seed microbiota.

In this study, we apply metabarcoding approaches to uncover the contribution of insect pollinators to the seed microbiota and to the transmission of seed-associated microorganisms. Since bees (*Apoidea*: *Anthophila*) are amongst the most important pollinators^34^ and harbour bacteria that are shared with flowers^12^, we have examined the effect of bee pollination on the seed microbiota of oilseed rape (OSR; *Brassica napu*s) by performing pollination exclusion experiments. Our results show that bee pollination participates in the microbial assembly of the seed by reducing the bacterial richness and diversity, increasing the variability amongst plants (beta dispersion) and introducing bee-associated taxa. Collectively, these data suggest that insect pollination is an ecological process involved in the assembly of the seed microbiota.

## Results

### Taxonomic composition of bee, pollen, nectar and seed microbiota

In this work, we performed community profiling analysis of the bacteria associated with bees, nectar, pollen, and seeds issued from: (i) bee pollination (BP seeds); (ii) autonomous self-pollination (ASP seeds) and iii) from a hand-pollinated sterile plant line (“male sterile” seeds, MS seeds) (Supp. Figure S1). Analysis of the amplicon sequence variants (ASV) taxonomic affiliation showed that *Proteobacteria* and *Firmicutes* are the dominant *phyla* in honey bee samples (Supp. Figure S2). These phyla contain the main taxa of the “core gut” microbiome of honey bee workers; *Frischella*, *Gillamella, Snodgrassella, Lactobacillus* and *Buchnera* spp^35^. In 2018, nectar samples were dominated by the genus *Acinetobacter*. This taxon was also found in high abundance in the seeds (see below).Concerning OSR pollen, samples were also dominated by *Firmicutes* and *Proteobacteria* in both years (Supp. Figure S2).

**Figure 1:**
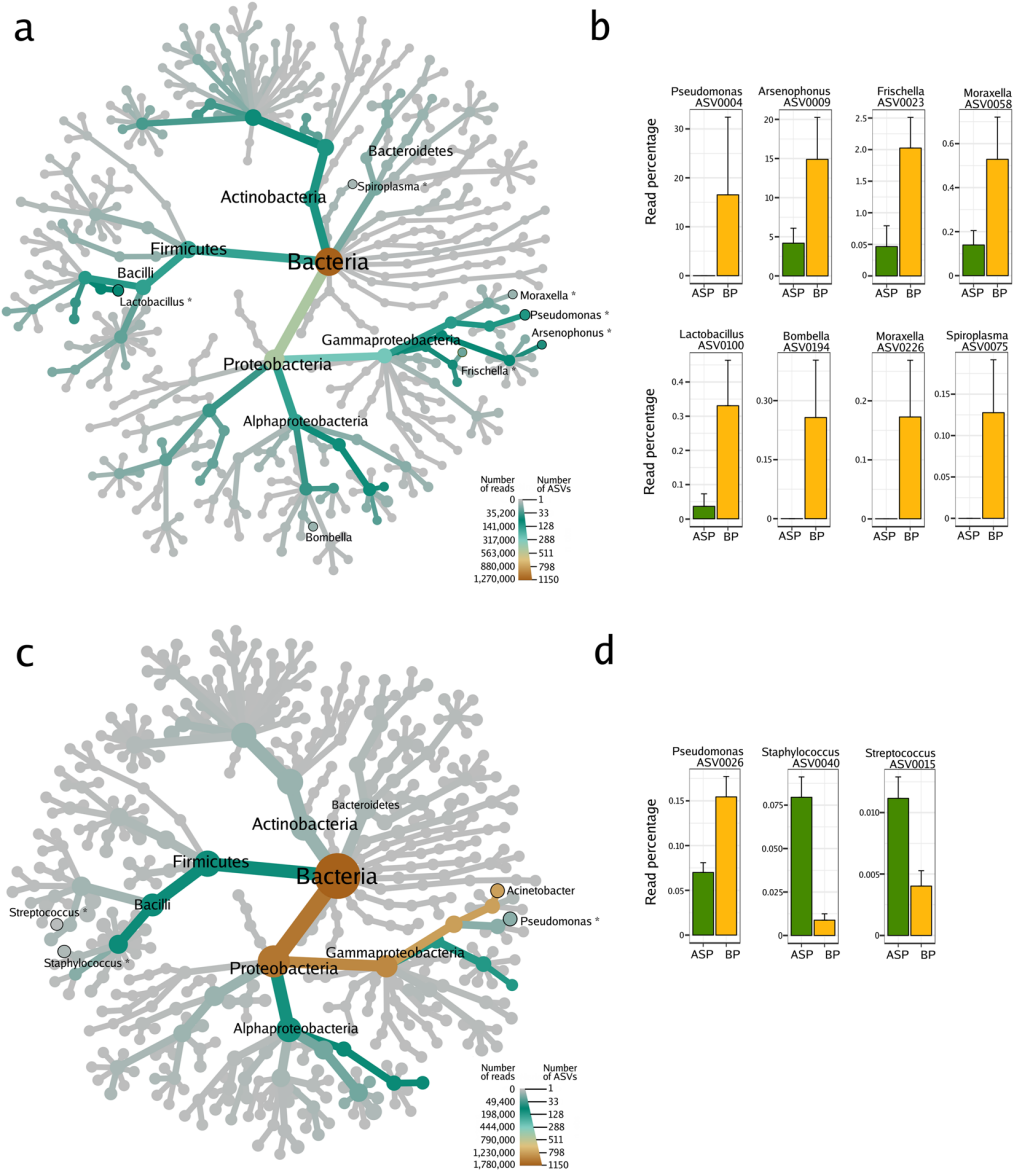
Microbial composition of oilseed rape seed samples issued from flowers exposed to honey bee pollination or autonomous self-pollination. Heat trees show the microbial composition of the seeds samples harvested in (**a**) 2017 and (**c**) 2018. The size of the nodes refers to the number of ASVs of known identity and the colour of the nodes and edges represent the ASV read abundance. Asterisks (*) indicate the taxonomic affiliation of ASVs with significant changes in relative abundance (according to Linear Discriminant Analysis Effect Size; LefSe) in relation to the pollination mode. ASVs with significant changes in relative abundance are displayed on the right part of the figure for both respective years ((**b**) 2017, (**d**) 2018). BP: honey bee-pollination; ASP: autonomous self-pollination.

In the case of microbial assemblages associated with seeds, the most common bacterial genera belonged to the *Actinobacteria*, *Bacteroidetes*, *Firmicutes* and *Proteobacteria* **(**Fig. 1a,c**)**, which is in agreement with other microbiome studies performed on OSR seeds^30^. Seed samples from 2018 were dominated by the genera *Acinetobacter* and *Pantoea* (around 80% of the reads were affiliated to these two genera; Fig. 1c). Pollination by honey bees changed (logarithmic LDA score higher than 2.5) the seed abundances of 3 ASVs, while mason bee pollination afffected the abundances of 15 ASVs (Fig. 1d; Supp. Tables 3 and 4).

In seed samples collected during 2017, *Sphingobium*, *Pseudomonas*, *Lactobacillus* and *Gillamella* were the most predominant genera. Honey bee pollination affected the relative abundances of 8 ASVs (Fig. 1b**;** Supp. Table 2). Interestingly, 4 of these taxa are specifically associated with honey bees and part of the bee core microbiota **(**Fig. 1a**)**; *Arsenophonus*^36^, *Frischella*^37^, *Spiroplasma*^38^ and *Lactobacillus*^39^ are bee-associated taxa and were more abundant in seeds issued from BP as opposed to those issued from ASP. ASV0075 showed 99% identity with the honey bee pathogen *Spiroplasma apis* strain B31^T^ ^38^ and ASV0100 showed 100% identity with *Lactobacillus mellis* strain H1HS38N, a symbiotic bacterium inhabiting the bee stomach^39^. The other two taxa enriched in BP samples belong to the genus *Moraxella*, a bacterial genus that is commonly found in nectar. The acetic acid bacteria of the genus *Bombella*, which are found in the gut of bumble bees (*Bombus* spp.) and honey bees^40^, were only present in the bees themselves and in the seeds issued from BP. Due to its low relative abundance, the *Bombella* ASV did not achieve the 2.5 fold change criteria. These results suggest that insect pollinators transfer bacteria (*Arsenophonus*, *Frischella*, *Spiroplasma*, *Lactobacillus* and *Bombella*) from the nectar collected or from the insect itself to the seed via the floral pathway.

### Seed microbial alpha diversity is modified by honey bee pollination

**Figure 2:**
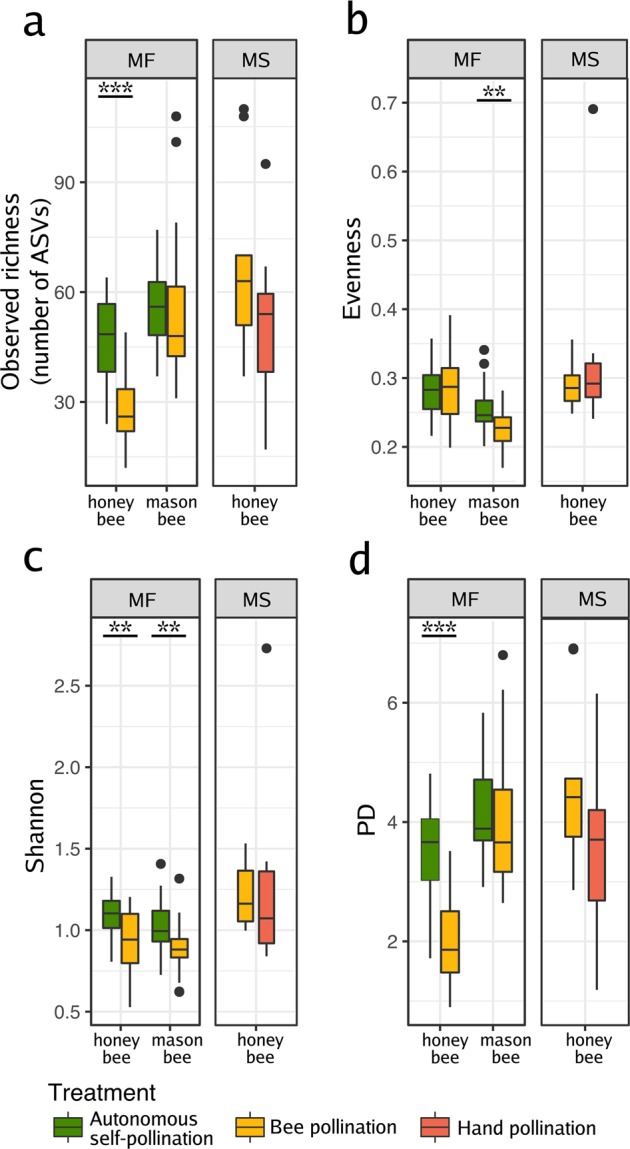
Changes in microbial richness and alpha diversity between seed samples. Observed richness (**a**), evenness (**b**) and diversity (Shannon and Faith’s PD phylogenetic diversity; (**c**,**d**) respectively). The indexes were estimated in seed samples harvested from oilseed rape male fertile (MF) plants pollinated by bees or left for autonomous self-pollination. Additional indexes were calculated from seed samples harvested from a male sterile (MS) line that was hand- or insect-pollinated. Wilcoxon rank-signed tests were performed to assess the effect of pollination on richness and alpha diversity. Asterisks denote statistically significant differences between conditions considered at *p-value* < 0.05 (*), *p-value* < 0.01 (**), or *p-value* < 0.001 (***). Richness, evenness and Shannon diversity were assessed with the number of ASVs rarefied at 12,000 sequences per sample.

To further investigate how bee pollination affects the structure of seed microbial assemblages, bacterial richness and diversity indexes were assessed for the seed-associated assemblages in all the different treatments (Fig. 2). To compare the alpha diversity in seed samples, non-parametric Wilcoxon rank-signed tests were performed on the rarefied data set. No statistical differences were found in alpha diversity in the 2017 seed samples (N = 11). In contrast, variations of ASV richness and diversity were observed between seed samples in relation to the pollination mode in 2018 (N = 53, Fig. 2a). Bacterial richness decreased in seeds issued from honey bee pollination (p < 0.0001; Fig. 2a). However, we do not observe the same trend in the seeds issued from mason bee pollination (Fig. 2a). Differences between these two types of pollinators likely reflect differences in the foraging intensity of the insects (see discussion section).

Seeds issued from hand pollination on the MS line (HP seeds) did not differ in bacterial richness from those produced from BP, suggesting that the decrease in richness may be partly explained by the amount of pollen deposited on the stigma and not by the selection of insect-associated bacterial taxa (Fig. 2a). We observed a reduction in the evenness in the samples issued from mason BP. Indeed, *Acinetobacter* is the dominant taxa in seeds issued from mason BP.

To determine the impact of bee pollination on the diversity of the seed microbiota, we calculated several diversity indexes. Bacterial diversity was lower in seeds issued from honey bee pollination (Shannon and Faith’s PD phylogenetic diversity; p = 0.003 and p < 0.0001 respectively; Fig. 2c,d). A similar trend with the seeds produced from mason bee pollination was also observed (Fig. 2c), where the Shannon index was also reduced (p = 0.001). All together, results suggest that bee pollination decreases microbial assemblage diversity in seeds.

### Effect of bee pollination on the structure of the seed microbiota

**Figure 3:**
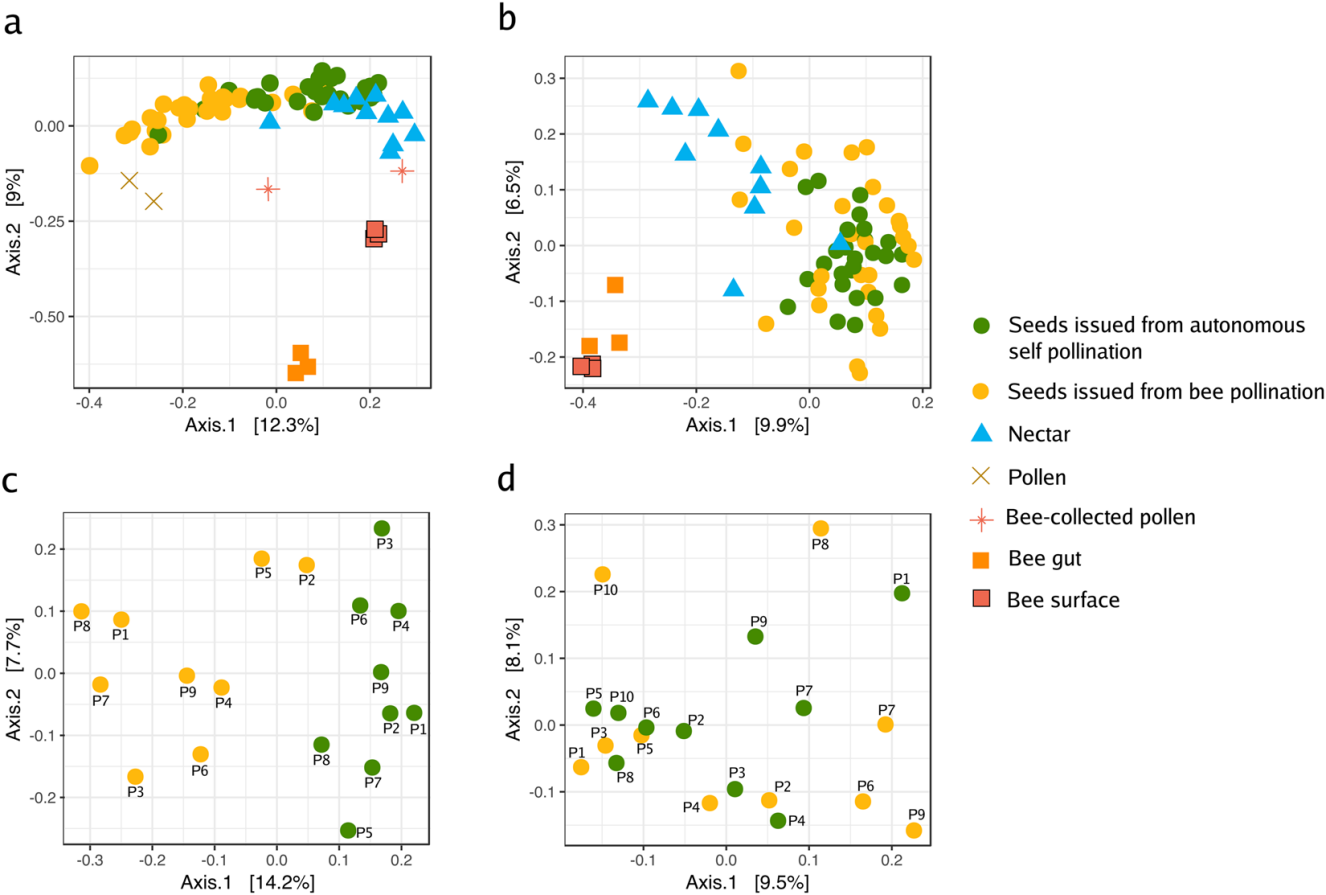
Ordination of unweighted UniFrac matrices with principal coordinate analysis (PCoA) showing variation in microbial composition in 2018. PCoA plots show the ordination of all samples from the honey bee (HB) (**a**), and the mason bee (MB) (**b**) experiment. Seed microbial assemblages of each plant (pooled samples) are represented in panel (**c**) for HB pollination, and in panel (**d**) for MB.

The similarity of community composition between samples was estimated through PCoA ordination of unweighted UniFrac distances (Table 1 and Fig. 3 for 2018 data). The relative contribution of pollination was investigated through canonical analysis of principal coordinates (CAP) followed by PERMANOVA (Table 1). Bacterial composition differed between plant materials (seeds, nectar and pollen; Fig. 3a); nectar and seeds clustered together in the ordination plot and separated from pollen. The type of plant material explained 35.5 and 9% of the variation in microbial composition in 2017 and 2018, respectively (Table 1; *p* = 0.0002 and *p* = 0.0001). As expected, bee-associated microbial assemblages were distinct than those associated with plant tissues. It is of interest that microbes associated with the surface of the highly social honey bees were different from the microbes living inside the bee gut^35^ and this was not the case for the solitary mason bee, that harboured similar microbial assemblages on its surface and gut (Fig. 3b).

**Table 1:** Results of the constrained analysis of principal coordinates.

Data set name	Year	Data set material	Explanatory variable	Samples	Proportion of constrained inertia (%)	F-value	*p*-value
All samples	2017 2018	ASP and BP seeds, nectar, pollen, bees	Year	198	**5.7**	**11.45**	**0.0001**
Experiment 2017	2017	ASP and BP seeds, nectar, pollen	Material	16	**35.54**	**3.584**	**0.0002**
Experiment 2017 male fertile line	2017	ASP and BP seeds	Pollination mode	10	NA	1.19	NS
Experiment 2017 male sterile line	2017	ASP and BP seeds	Pollination mode	12	NA	1.073	NS
Experiment 2018	2018	ASP and BP seeds (honey bee and mason bee), nectar, pollen	Tunnel	134	**4.5**	**6.22**	**0.0001**
Honey bee experiment 2018	2018	ASP and BP seeds, nectar, pollen	Material	69	**9.3**	**3.28**	**0.0001**
Honey bee experiment 2018 male fertile line	2018	ASP and BP seeds	Plant ID	18	NA	0.91	NS
Honey bee experiment 2018 male fertile line	2018	ASP and BP seeds	Pollination mode	18	**12.3**	**2.23**	**0.001**
Honey bee experiment 2018 male sterile line	2018	ASP and BP seeds	Pollination mode	21	NA	1.29	NS
Mason bee experiment 2018 male fertile line	2018	ASP and BP seeds	Plant ID	20	NA	1.33	NS
Mason bee experiment 2018 male fertile line	2018	ASP and BP seeds	Pollination mode	20	NA	1.14	NS

Proportion of variance explained by the indicated variable on the different data sets. The proportion of constrained inertia, F-values and *p*-values were calculated through a canonical analysis of principal coordinates followed by PERMANOVA. Seeds issued from autonomous self-pollination and bee pollination are referred to as ASP and BP seeds, respectively.

Microbial composition of the seeds did not reflect pollination mode in 2017 (Table 1). However, in 2018, flowers pollinated by honey bees produced seeds with changes in microbial composition compared with those from ASP seeds, showing that honey bee pollination may affect the seed microbiota composition (Fig. 3c). Moreover, BP seed microbial assemblages diverged from those of ASP seeds and were closer to those of pollen (Fig. 3a). According to CAP analyses, honey bee pollination explained 12.3% of the variation in bacterial community composition in the seed samples in 2018 (*p* = 0.001). In 2017, we observed a similar trend, although the change was not statistically significant due to the small sample size (Table 1). In contrast, the seed microbial composition did not change due to mason bee pollination, probably due to their reduced visitation rates recorded during our experiment.

In both years, the bacterial composition of the seeds produced by honey bee pollination did not differ from that of HP seeds on the MS line (Table 1). Segregation between the MS seeds from the honey bee and the hand pollination treatments was not observed in the ordination plots (data not shown). In agreement with these results, the constrained analysis of principal coordinates supported that the pollination mode of the MS line did not affect seed-associated microbial assemblages. Thus, the differences observed between seeds that resulted from honey bee pollinated flowers and seeds that came from ASP flowers was due to the amount and the varied origin of the pollen, and not necessarily to the insect associated taxa. This suggests that, whichever vehicle transports the pollen, the critical aspect is the origin and amount of pollen.

### Pollination by honey bees increases microbial beta dispersion among seeds

**Figure 4:**
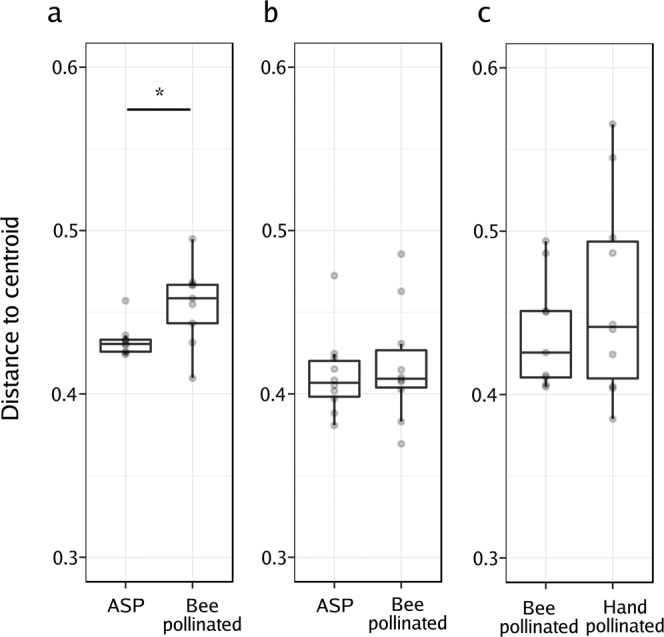
Effect of the pollination mode on the structure of seed microbial assemblages. Analysis of the multivariate homogeneity of group dispersions (variances). Boxplots represent the distance to the centroid of seed-associated microbial communities of male fertile (**a**,**b**) and male sterile plants (**c**) submitted to different modes of pollination. (**a**) Distance to the centroid of seeds issued from male fertile plants subjected to autonomous self-pollination (ASP) or honey bee pollination, (**b**) distance to the centroid of seeds issued from male fertile plants subjected to ASP or mason bee pollination, (**c**) distance to the centroid of seeds issued from male sterile plants subjected to honey bee pollination or hand-pollinated with pollen of different plants. Wilcoxon signed-rank tests were performed to assess the effect of the pollination mode on the distance to the centroid. Asterisks denote statistically significant differences between conditions considered at *p-value* < 0.05 (*).

Following honey bee pollination, bacterial assemblages associated with BP seeds were more variable than those of the ASP seeds (Fig. 3c). In this experiment, two pollination strategies were represented within each plant, to permit comparison of the variability in the bacterial composition of seeds. Beta dispersion was assessed by comparing the distances in the principal coordinate space (created using the unweighted UniFrac distances) to the group (pollination mode) centroid^41,42^. In this way, we assessed the beta dispersion as the variability of the ASV composition at the plant level (inter-plant variation, n = 9, n = 10 & n = 10 for honey bee, mason bee and HP on the MS line, respectively). The beta dispersion of ASP and BP seed microbial assemblages in the mason bee treatment did not differ, nor between honey bee and hand pollinated seed microbial assemblages in the MS line. However, the beta dispersion was significantly higher in seed communities that were exposed to honey bee pollination in contrast to those that came from ASP (Wilcoxon rank-signed test, *p* = 0.02; Fig. 4).

## Discussion

Little is known about neutral or niche-based ecological processes involved in the assembly of the seed microbiota. Niche processes, like selection by the environment, shape the structure of seed-associated fungal assemblages^25^. Furthermore, host-filtering processes are involved in the seed microbiome assembly of oilseed rape^30^ and tomato (*Solanum lycopersicum*)^23^. On the other hand, neutral processes related to ecological drift are important drivers of the seed bacterial communities structure in radish (*Raphanus sativus*)^28^. Together, these pioneering studies suggest that seed-associated microbial communities consist of a few dominant taxa that are probably niche-selected and multiple scarce taxa the distributions of which could be explained by neutral processes. The present study supports the hypothesis that insect pollination is a neutral ecological process of microbial dispersal between flowers and seeds and influences the assembly of the seed microbiota. Our results show that *Apis* pollination can change the diversity of seed-associated bacterial assemblages and, in some cases, introduce bee-associated taxa.

The proximity between a floral structure and the developing seed suggests that microbes associated with the flower could, eventually, colonize the developing seeds through the floral pathway. Thus, the mechanisms that disperse microbes onto flowers (i.e. insect pollination) could have important implications for the transmission of non-specialized microbes to the developing seeds. Previous studies have shown that insect pollination modifies the composition of microbial assemblages associated with flowers^9^, nectar^7,8,42^ and pollen^11^; however, little is known about the effect of insects pollinators on seed microbial communities. According to our data, changes in seed microbiota were mostly associated with honey bees (*A. mellifera*) while mild effects were observed following pollination by the red mason bee (*O. bicornis*). These two bee species possess different microbiota, reflecting their life histories with *Apis* being a social bee and *Osmia* a solitary bee. Microbiota of *Apis* guts have low taxonomic diversity, with very similar microbial phylotypes between individuals, constituting a bee core microbiota^35^. In contrast, solitary bees do not harbour this core bee microbiota. The most common bacteria in solitary bee species are a widespread phylotype of *Burkholderia* and *Wolbachia*^43^. The social behaviour of *Apis* species facilitates microbial transmission among bees and is key to maintaining a consistent gut microbiota^35,37,43^. Thus, the potential transmission by microbes to the seed of solitary bees is more random than the transmission of microbes by social bees where some members of the core bee microbiota would be systematically transmitted. In this study, the forager density between the two bee species differed considerably and we observed a strong difference in visitation rates. Indeed, honey bees visited the flowers more intensively (ca. 1 visit per flower every 2 min), compared to the mason bees (ca. 1 visit per flower every 10 min). Moreover, while the honey bee colony used in these experiments consisted of a population of ca. 5000 worker bees with a population of foragers of at least 1000 individuals, only 200 female and male cocoons of mason bees were used in this study. It is also noteworthy that the visits to the experimental flowers during the mason bee experiment were conducted mostly by males that were searching for nectar between matings. These differences are reflected in the resulting seed microbiota, suggesting that the amount of contact between the insect and the flower may be a determining factor in the effect of the insect pollinator on the seed microbiota. Indeed, the time that a bee spends foraging on a flower is correlated with the transmission of bee pathogens^6^. The potential role that colony size, sociality and foraging behaviour (especially forager density and flower handling time, i.e. time spent per flower) has on the seed microbiota should be assessed to shed light on the role that different pollinators have in the transmission of seed-borne diseases^10,14^.

The microbial assemblages associated with seeds examined in this study are dominated by members of the *Actinobacteria*, *Bacteroidetes*, *Firmicutes* and *Proteobacteria* phyla **(**Fig. 1**)**. These phyla were also prevalent in other studies of the oilseed rape seeds^30^. Yet, the taxonomic profiles we observed were different between the two years of this study. This is not so surprising since different factors (such as climatic conditions) have a strong influence on the seed microbiota^25^. *Acinetobacter* was the dominant genus of seeds and nectar in 2018. This is a widespread genus that occurs in animals, plants and the environment^44^. In plants, *Acinetobacter* spp. have been described as plant-growth-promoting bacteria^45^. In our 2018 experiments, this taxon was already present in pollen and nectar prior to the pollinator exclusion experiments. It is, thus, possible that *Acinetobacter* acted as a pioneer taxon in seeds exerting a strong priority effect in the final microbial composition of the seed.

While mason bees carry pollen in its abdominal scopa, the honey bee aggregates pollen grains by adding regurgitated nectar or diluted honey to transport it to its nest on the corbiculae of its hind legs. Once packed into the corbiculae, pollen grains are no longer available for pollination, nonetheless, some pollen grains remain on other bee surfaces were they can be deposited onto the stigma. The addition of nectar raises the humidity of the pollen grain, causing it to swell and expose the intine^46^. The taxonomic profiles of the 2017 seed samples show that honey bee pollination changed the seed microbiota by increasing the abundance of bee- or nectar-associated taxa. It is then tempting to speculate that the way the honey bee processes pollen allows for the transmission of bee-associated bacteria to the seed. Indeed, *Spiroplasma*, *Lactobacillus*, *Arsenophonus*, *Frischella* and *Bombella* are insect-associated symbiotic bacteria living in the bee gut^36,37,47,48^ and were more abundant and, in some cases, exclusively occurred only in seeds issued from BP as opposed to ASP. These results illustrate the possibility of insect-transmitted bacteria colonizing the seed. This begs the questions “*could plants act as reservoirs for bee symbionts and pathogens?”* and *vice versa* “*could bees act as reservoirs for plant symbionts and pathogens?”*.

One mechanism by which plants may increase fitness is through selection of beneficial members of the seed microbiome that could enter via pollinators; however, the persistence of these members and their roles in determining plant fitness is unknown. Most of the bee-associated taxa recovered in bee-pollinated seeds (*Arsenophonus*, *Frischella*, *Spiroplasma* and *Lactobacillus)* are bee symbionts that are not adapted to the seed environment and will probably not be selected in seedlings. Nonetheless, some of the other taxa that are increased in BP seeds (e.g., *Pseudomonas*; Fig. 1b–d) may be able to act as a plant-growth promoting bacteria, with a positive effect on seed germination, seed viability and in general, plant fitness. Bee pollination services can enhance seed production in quantity and quality^49^. Insect pollination of OSR modifies functional characteristics, such as flower timing & effort, plant size & shape, pod production, root biomass, which increase seed production and quality^50^. It is unknown if these benefits could be, in part, the consequence of a microbial exchange between the plant and the insect. Future experiments monitoring the dynamics of the microbiota bee-pollinated seeds during seed emergence could elucidate the resilience of these insect-transmitted microbial taxa in the plant cycle and their effect on plant yield.

In our experimental design, the effect of honey bee pollination on seed microbiota was assessed in two different plant genotypes: a male fertile line and a male sterile line that does not produce pollen. Seed microbial assemblages of seeds issued from honey bee-pollination of the male sterile plant line did not differ from hand-pollination. This suggests that the diverse origin (many source plants) and/or copious amount of pollen delivered either by a honey bee or by a paintbrush have similar effects on the seed microbiota. In contrast, honey bee pollination did have an effect as compared to the reduced amount and single origin of the pollen of ASP. The amount of pollen delivered to the stigma impacts the germination rates of pollen grains, with small pollen populations germinating poorly. This population effect is, partly, explained by the availability of certain growth factors, such as calcium ions^51^, flavonols^52^ and phytosulfokine-alpha^53^. The greater availability of nutrients presented in large pollen populations might also provide nutrients for bacteria. The availability of nutrients fosters competition between the microbial taxa, which could explain the observed decrease in species richness observed in honey bee-pollinated seeds.

Alternatively, the period that the stigma is receptive may also affect the alpha diversity. Bacteria are generally unable to actively penetrate plant tissues and rely on openings, such as wounds, stomata, lenticels and nectarhodes (stomate-like openings on the nectary)^54^. The stigma surface provide a potential route by which bacteria can penetrate the seed^14,17^, possibly occurring during the penetration of the pollen tubes. Following flower opening, oilseed rape flowers require on average ~13 h of exposure to pollinators to complete their sexual functions^55^. Since the removal of pollen triggers flower senescence^55^, flowers visited by bees were likely to have senesced faster than the flowers left to self-pollinate autonomously. The increased flower longevity in the ASP treatment may have facilitated the entry of bacteria, explaining the higher diversity of the seed microbial communities. Future experiments should aim at disentangling how insect pollination and flower longevity affect the seed microbiome.

Honey bee pollination enhances the variation in the structure of seed bacterial assemblages (Fig. 4a). This result is in agreement with the findings of Vanette *et al*.^42^ who reported that pollinators increase dispersal of microorganisms and, ultimately, enhance dissimilarity between nectar microbial assemblages. These findings suggest a high stochasticity in the order in which bacterial species arrive (stochasticity of microbial dispersal), and due to strong priority effects, the composition of the assemblages can diverge^42^. Pollination by *Apis* would, consequently, favour the arrival of new species to the flower and seed, increasing the variability of the seed-associated microbial communities. Our study will serve as a foundation for future experiments that directly target the impact of priority effects on beta dispersion and on the assembly of the seed microbial communities.

## Conclusions

This study aimed to uncover the contribution of bee pollination to the seed microbiota. We have found differences in richness, diversity and species composition in the microbial community of seeds issued from bee pollination, as compared to those from flowers with autonomous self-pollination. Our findings with two different bee species suggest that foraging behaviour (foraging rates/intensity) mediates the effect of the insect-pollinator on the seed microbiota. Additionally, the amount and origin of the pollen may also shape the microbial assembly. These results provide novel insights about determinants involved in the transmission of bacteria from flower to seeds, and have important implications in re-evaluating pollinators services, which should include microbe transfer to the seeds.

## Materials and Methods

The effect of bee pollination on OSR seed-associated microbial assemblages was assessed during two years (2017 and 2018) on two plant lines: (i) a male fertile F_1_ hybrid (MF; cv ‘Exocet’) that was bagged to exclude insects and be autonomously self-pollinated or left open to bee visits from two pollinator species, the domestic honey bee (*Apis mellifera)* or the red mason bee (*Osmia bicornis)*; and (ii) its male sterile parent, thereafter referred to as the MS line, that does not produce pollen and was either bagged and hand-pollinated with pollen from many different plants of the MF line, or exposed to honey bee visits and pollination. In total, 198 samples corresponding to bees, nectar, pollen and seeds were analyzed by 16 S rRNA gene amplicon Illumina sequencing **(Supp**. Table 1).

### Pollinator exclusion experiments

Pollinator exclusion experiments were performed inside two 22 × 8 m insect proof tunnels at the INRA research station in Avignon during 2017 and 2018. Inside the tunnels, seeds of oilseed rape (*Brassica napus*) were sown in the soil in four 18 m long rows (20 plants per row). Two winter oilseed rape lines were sown side by side: a male fertile F_1_ hybrid line ‘Exocet’ and its male sterile parent which does not produce pollen. Winter oilseed rape was chosen because it is a highly self-fertile plant that produces nectar attractive to bees, and because a male sterile line was available. Plants were watered twice a day with an automated water drip system.

In 2017, the pollinator exclusion experiment was only carried out using honey bees on 5 plants of the male fertile line in a single tunnel. In 2018, the pollinator exclusion experiments included 1) honey bees on the male fertile line in one tunnel 2) mason bees on the male fertile line in a second tunnel and 3) honey bees on the male sterile line in the first tunnel. For each experiment, ten plants were chosen as experimental plants based on their homogeneous appearance. On each plant, six flowering branches were marked using flower markers of two different colours (three panicles each) and bagged with hydrophilic plastic bags made of osmolux film (Pantek France, www.pantek-france.fr/agriculture.html). The osmolux bags are gas-permeable, but prevent all contact with insects, even small ones such as thrips (Thysanoptera)^56^. On the day of the introduction of the bees into the tunnels, three color-coded branches were un-bagged and exposed to bee visits (bee pollination treatment), while the three others remained covered (autonomous self-pollination treatment). Bees were allowed to forage freely in the tunnel amongst experimental and non-experimetnal plants. For the male sterile line, the panicles that remained bagged during the introduction of the bees were then hand-pollinated using a fine paint brush with pollen collected and pooled from many flowers (>200) from several plants of the male fertile line (>10). After 48 hrs, the bees were removed from the tunnels and the uncovered branches were re-bagged. All branches were kept bagged for an additional 48 hours to ensure that no bee was left in the tunnels, at which point all bags were removed.

In order to compare between bee pollinators, one tunnel was used to perform experiments using honey bees (*Apis mellifera*) where a 5-frame hive (adult worker population ~5000) was introduced. In the other tunnel, 100 male and 100 female cocoons of the red mason bee (*Osmia bicornis*) were introduced. Male mason bees were highly active visiting flowers and mating with the females during the experiment. The female mason bees were mostly inactive after mating and due to our experimental design, they were removed from the tunnel before they started provisioning their nests.

### Material collection

Prior to the introduction of bees into the tunnels, pollen (N = 2) and nectar (N = 20) samples were collected from bagged flowers and kept at −20 °C until DNA extraction. Pollen was collected by dissecting closed flower buds and separating the anthers. Anthers were left to dehisce for 4 h at room temperature in glass Petri dishes. To recover the pollen, the dried anthers were placed in a steel tea ball and vibrated using a Vibri Vario pollinator. Nectar was collected with 2 µl capillary tubes between the base of the anthers and transferred to 2 ml Eppendorf tubes. During the experiment, honey bee foragers were captured and their pollen loads removed and frozen for further analysis (N = 6). Once mature (2 months after pollination), OSR fruits (siliques) were collected in large paper bags and taken into the laboratory. Under aseptic conditions, seeds were removed from the siliques and placed in small paper bags. Seeds from siliques in the same raceme that had received the same treatment were pooled to create 0.5 g samples.

### DNA extraction, amplicon library construction and sequencing

For seed sample preparation, a total of 0.5 g of oilseed rape seeds of each sample were transferred to sterile tubes containing 2 ml of PBS supplemented with 0.05% (vol/vol) of Tween 20. Samples were incubated for 2 h and 30 min at 4 °C under constant agitation (150 rpm). In the case of bee samples preparation, to obtain bee surface microbial assemblages, bees were sonicated in 1 ml of PBS buffer with 0.05% Tween 20. After removing the liquid, insect samples were re-suspended in 1 ml of PBS and crushed to recover the microbes inside the bees. All the suspensions were centrifuged (12,000×*g*, 20 min, 4 °C) and pellets were stored at −20 °C until DNA extraction. Total DNA extraction was performed with the PowerSoil DNA isolation kit (MoBio Laboratories) using the manufacturer’s protocol.

Amplification, purification and pooling for amplicon library construction were conducted following the protocol described in Barret *et al*.^24^. Briefly, for amplicon construction, two rounds of PCR were performed. The first round was designed to target the region V4 of the 16 S rRNA with the PCR primers 515 f/806s^57^. All PCR amplifications were performed with a high-fidelity polymerase (AccuPrime *Taq* DNA polymerase; Invitrogen) according to the manufacturer’s protocol and 10 μl of DNA suspension. After amplicon purification, a second round of amplification was performed with 5 μl of purified amplicons and primers containing the Illumina adaptors and indexes. All amplicons were purified, quantified and pooled in equimolar concentrations. Finally, amplicons libraries were mixed with 10% PhiX control according to Illumina’s protocols. Two sequencing runs were performed in this study with MiSeq reagent kit v2 (500 cycles) for the samples of 2017 and MiSeq reagent kit V3 (600 cycles) for the samples of 2018.

### Data analysis

MiSeq runs were analysed separately. Primers sequences of fastq files were first cut off using Cutadapt 1.8^58^. Files were then merged and processed with DADA2 v.1.8.0^59^ according to the recommendations of the workflow “DADA2 Pipeline Tutorial”. The workflow was modified in the truncLen parameter to adjust it to the quality of the sequencing run. The 16 S rRNA amplicon sequence variants (ASV) resulting from DADA2 were aligned with a naive Bayesian classifier against the Ribosomal Database Project training set 16 database. Statistical analyses were done with Rstudio v3.3 using the R package *phyloseq* v1.24.2^60^. The *Metacoder* R package v 0.3.0.1^61^ was used to plot the distribution of ASV, associated with a taxonomic classification in heat trees. Observed taxa richness, evenness, and diversity were calculated on a rarefied dataset at 12,000 reads per sample and differences were assessed by Wilcoxon signed-rank tests. Variances in community composition between the different samples were assessed by unweighted UniFrac distance^62^. Principal coordinate analysis (PCoA) was used for ordination of UniFrac distances. Permutational multivariate analysis of variance (PERMANOVA)^63^ was calculated to investigate the effect of pollinators on microbial community profiles as implemented by the package *vegan* v2.5–3 in R. Moreover, to quantify this contribution, a canonical analysis of principal coordinates was performed with the function *capscale* of the *vegan* package. Changes in relative abundance of ASV between the different seed samples were determined using linear discriminant analysis (LDA) effect size using the LefSe tool^64^ available at http://huttenhower.sph.harvard.edu/galaxy.

To compare the beta dispersion amongst seeds resulting from the different pollination modes, the variability in ASV composition was quantified within each pollination treatment using the betadisper function in the *vegan* package in R. Beta dispersion is measured by the distance to the centroid of each treatment group in the principal coordinate space^41^.

## Supplementary information

Supplementary Figures.

Supplementary Tables.

## Data Availability

The raw sequencing data is available at the European Nucleotide Archive (ENA) under the study accession PRJEB31847. Tables and scripts used in this work are publicly available in GitHub.
