# Supplementary material for: Insect pollination: an ecological process involved in the assembly of the seed microbiota
Source: Sci Rep. 2020 Feb 27;10:3575. doi: 10.1038/s41598-020-60591-5 (PMC7046713; doi:10.1038/s41598-020-60591-5)

Supplementary Information

Insect pollination: an ecological process involved in the assembly of the seed microbiota

Alberto Prado[1]*, Brice Marolleau[2], Bernard Vaissière[3], Matthieu Barret[2], Gloria Torres-Cortes[2]*[ß]

*These authors contributed equally to the work

[ß]Correspondence to gloriatorresco@gmail.com

[1]Escuela Nacional de Estudios Superiores, Unidad Juriquilla, UNAM, Querétaro, México

[2]INRA, UR 406 Abeilles et Environnement, F 84914, Avignon, France

[3]IRHS, Agrocampus-Ouest, INRA, Université d'Angers, SFR4207 QuaSaV, 49071, Beaucouzé, France

Supplementary Figures

Supplementary Figure S1. Rarefaction curve. Representation of the observed number of ASVs as a function of the sample size.

Supplementary Figure S2. Heat trees showing the taxonomic composition of different material collected in 2017 (left part of the panel) and 2018 (right part of the panel). Microbial composition of honey bees (a,b), pollen (c,d) and nectar (e,f) is shown in color (grey branches represent absent taxa). Size of nodes refer to the number of ASVs of known identity and the color of nodes and edges represent the ASV read abundance

Supplementary Figure S1. Rarefaction curve. Representation of the observed number of ASVs as a function of the sample size

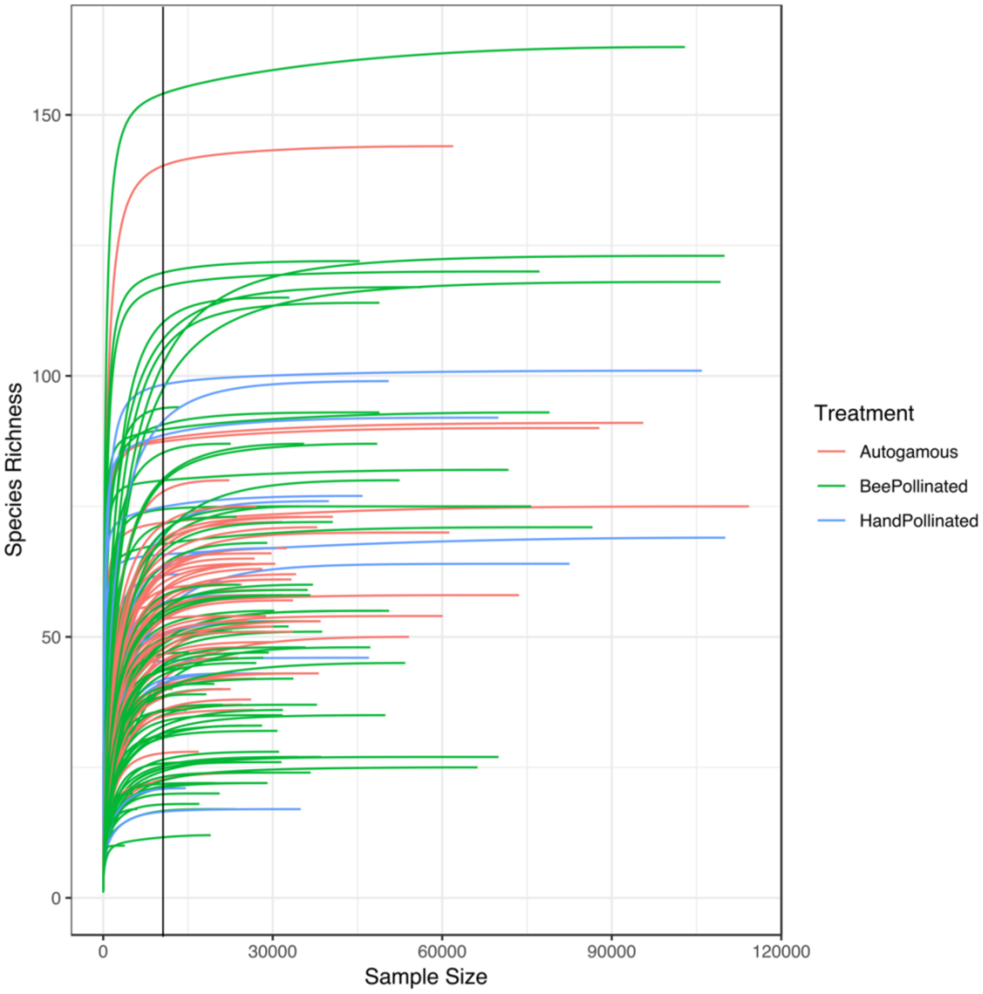

Supplementary Figure S2. Heat trees showing the taxonomic composition of different material collected in 2017 (left part of the panel) and 2018 (right part of the panel). Microbial composition of bees (a,b), pollen (c,d) and nectar (e,f) is shown in color (grey branches represent absent taxa). Size of nodes refer to the number of ASVs of known identity and the color of nodes and edges represent the ASV read abundance

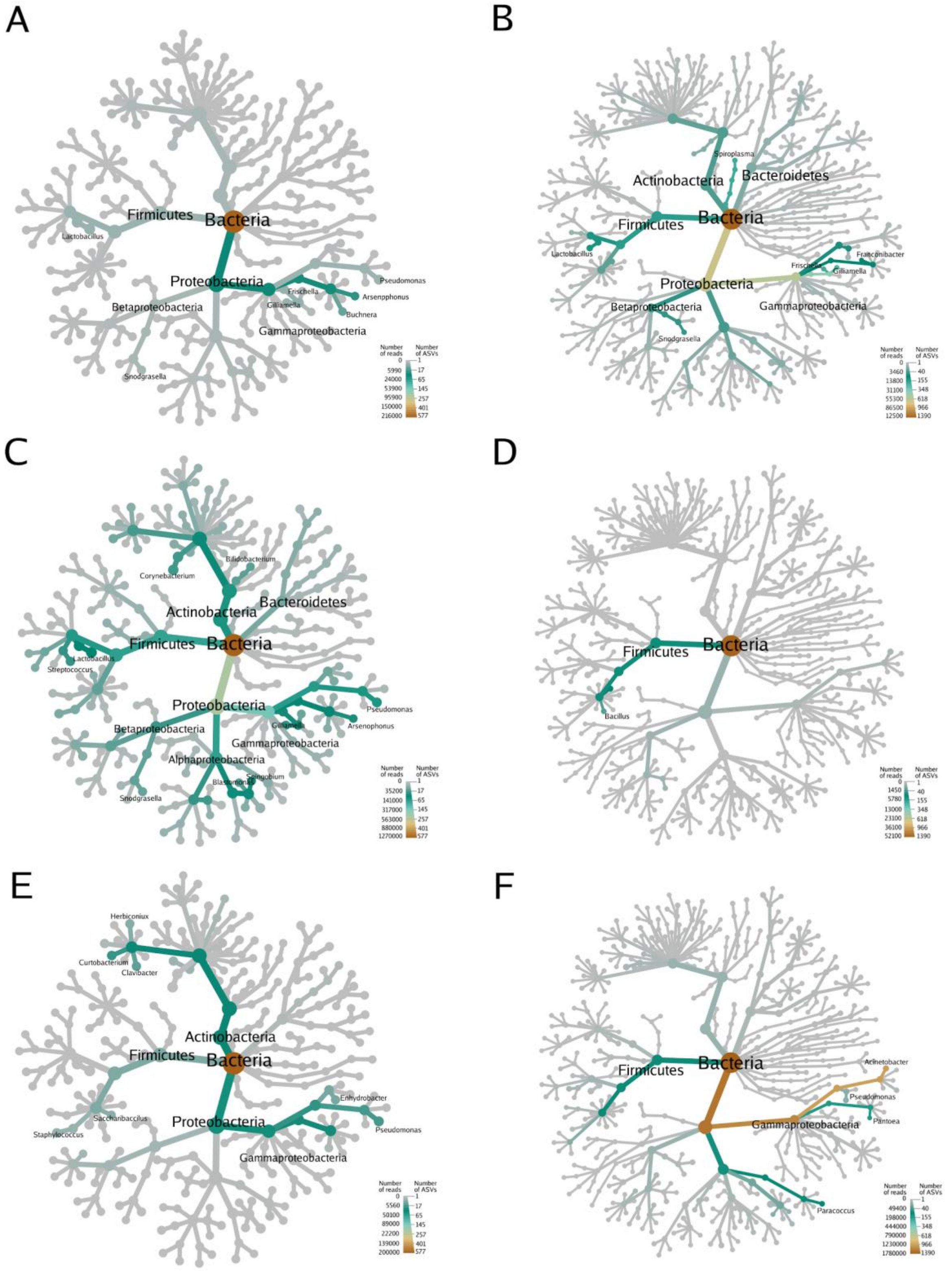

Supplement: Supplementary file 1 — Supplementary Figures. [file 41598_2020_60591_MOESM1_ESM.pdf]
